# Targeting Neutrophils to Treat Acute Respiratory Distress Syndrome in Coronavirus Disease

**DOI:** 10.3389/fphar.2020.572009

**Published:** 2020-10-09

**Authors:** Chih-Chao Chiang, Michal Korinek, Wei-Jen Cheng, Tsong-Long Hwang

**Affiliations:** ^1^Graduate Institute of Clinical Medical Sciences, College of Medicine, Chang Gung University, Taoyuan, Taiwan; ^2^Puxin Fengze Chinese Medicine Clinic, Taoyuan, Taiwan; ^3^Graduate Institute of Natural Products, College of Medicine, Chang Gung University, Taoyuan, Taiwan; ^4^Research Center for Chinese Herbal Medicine, Research Center for Food and Cosmetic Safety, and Graduate Institute of Health Industry Technology, Chang Gung University of Science and Technology, Taoyuan, Taiwan; ^5^Department of Biotechnology, College of Life Science, Kaohsiung Medical University, Kaohsiung, Taiwan; ^6^School of Traditional Chinese Medicine, College of Medicine, Chang Gung University, Taoyuan, Taiwan; ^7^Center for Traditional Chinese Medicine, Chang Gung Memorial Hospital, Taoyuan, Taiwan; ^8^Department of Anesthesiology, Chang Gung Memorial Hospital, Taoyuan, Taiwan; ^9^Department of Chemical Engineering, Ming Chi University of Technology, New Taipei City, Taiwan

**Keywords:** coronavirus disease 2019, severe acute respiratory syndrome coronavirus 2, acute respiratory distress syndrome, neutrophils, neutrophil extracellular trap

## Abstract

This review describes targeting neutrophils as a potential therapeutic strategy for acute respiratory distress syndrome (ARDS) associated with coronavirus disease 2019 (COVID-19), a pandemic caused by severe acute respiratory syndrome coronavirus 2 (SARS-CoV-2). Neutrophil counts are significantly elevated in patients with COVID-19 and significantly correlated with disease severity. The neutrophil-to-lymphocyte ratio can serve as a clinical marker for predicting fatal complications related to ARDS in patients with COVID-19. Neutrophil-associated inflammation plays a critical pathogenic role in ARDS. The effector functions of neutrophils, acting as respiratory burst oxidants, granule proteases, and neutrophil extracellular traps, are linked to the pathogenesis of ARDS. Hence, neutrophils can not only be used as pathogenic markers but also as candidate drug targets for COVID-19 associated ARDS.

## Introduction

Severe acute respiratory syndrome coronavirus 2 (SARS-CoV-2), an enveloped, nonsegmented, positive-sense RNA β-coronavirus, is the cause of the ongoing coronavirus disease 2019 (COVID-19) pandemic (Guo et al., 2020). SARS-CoV-2 is primarily transmitted by respiratory droplets and airway secretions through close contact with infected individuals (Lee and Hsueh, 2020). The main manifestations of COVID-19 are fever, cough, dyspnea, sore throat, fatigue, diarrhea (Guan et al., 2020), headache, nausea, vomiting (Li Y. C. et al., 2020), anosmia (loss of smell), and ageusia (loss of taste) (Vaira et al., 2020). Severe complications include acute respiratory distress syndrome (ARDS), septic shock, coagulation dysfunction, and multiple organ failure (Wang et al., 2020). The elderly (>65 years of age) and individuals with underlying secondary diseases, such as chronic obstructive pulmonary disease (COPD), cardiovascular disease, hypertension, and diabetes mellitus, tend to have severe complications and higher mortality rates (Yang et al., 2020). An effective therapy for COVID-19 remains under investigation (Lu, 2020).

Neutrophils are pivotal effector cells in the innate immune defense against infections in humans. Neutrophils migrate to infected tissues in multiple ways including rolling, adhesion, crawling, and transmigration. Subsequently, they are activated and exert inflammatory responses, such as phagocytosis, respiratory burst with superoxide anion production, degranulation with protease release, and NETosis with neutrophil extracellular trap (NET) formation (Liew and Kubes, 2019). Neutrophil inflammatory responses may be considered a double-edged sword; although they protect against infection, they also cause severe tissue damage. Activated neutrophils are involved in many acute and chronic inflammatory diseases as well as autoimmune disorders, such as respiratory diseases (ARDS, COPD, and asthma), cardiovascular diseases (atherosclerosis and thrombosis) (Németh et al., 2020), gastrointestinal diseases (inflammatory bowel disease and autoimmune hepatitis) (Honda and Kubes, 2018), neurological diseases (multiple sclerosis and Alzheimer’s disease) (Dong et al., 2018; Woodberry et al., 2018), skin diseases (psoriasis and Behçet’s disease) (Safi et al., 2018; Chiang et al., 2019), and metabolic diseases (diabetes mellitus and obesity) (Talukdar et al., 2012; Brotfain et al., 2015).

During the incubation period and nonsevere stage of COVID-19, the host immune system successfully destroys the virus and protects against disease progression. However, in the severe stage, SARS-CoV-2 replicates rapidly and causes massive tissue damage, particularly in the lungs. Thereafter, the destroyed cells cause a dysregulated inflammatory response and cytokine storm, leading to ARDS and other severe complications (Shi H. et al., 2020). Therefore, therapeutic strategies targeting hyperactivated neutrophils may be useful for treating COVID-19 associated ARDS. It has been suggested that a combination of antiviral and anti-inflammatory therapies effectively inhibit SARS-CoV-2 activity and reduce dysregulated immune reactions in COVID-19 (Stebbing et al., 2020).

In this review, we describe the roles of neutrophils in COVID-19 associated ARDS and provide an overview of suitable therapeutic strategies for targeting neutrophils. The particular focus is on clinical drugs and clinical trial drugs shown to affect neutrophil function (**Table 1**).

**Table 1:** Drugs targeting neutrophils.

Drug	Neutrophil Target	Clinical Stage	Approved Indication (Approved target)	Clinical Trial (Disease, Phase)	Clinical Trial for COVID-19 (Phase)^1^	Reference
**Sivelestat (Elaspol, ONO 5046)**	**NE**	Korea and Japan-approved	Acute lung injury, ARDS (elastase)			(Aikawa and Kawasaki, 2014)
**Alvelestat (AZD9668)**	**NE**	Clinical trial		NCT03636347 (COPD, phase 2)		(Barnes et al., 2020)
**BAY 85-8501**	**NE**	Clinical trial		NCT01818544 (non-CF bronchiectasis, phase 2)		(Watz et al., 2019)
**Lonodelestat (POL6014)**	**NE**	Clinical trial		NCT03748199 (CF, phase 1)		(Barth et al., 2020)
**CHF6333**	**NE**	Clinical trial		NCT03056326, NCT04010799 (non-CF bronchiectasis, phase 1)		(Gramegna et al., 2017)
**Elafin**	**NE**	Clinical trial		NCT02944279 (ARDS, phase 1)		(Barnes et al., 2020)
***N*-Acetylcysteine**	**Respiratory burst and ROS**	FDA-approved	Mucolytic (glutathione synthetase)		NCT04419025 (phase 4) NCT04455243 (phase 3) NCT04374461 (phase 2) and 3 more	(Allegra et al., 2002; Zhang et al., 2017; Andreou et al., 2020)
**Brensocatib (AZD7986)**	**DPP1**	Clinical trial		NCT03218917 (non-CF bronchiectasis, phase 2)	EudraCT 2020-001643-13 (phase 3)	(Palmér et al., 2018)
**Dipyridamole**	**PDEs**	FDA-approved	Prevention of postoperative thromboembolism, stroke (PDEs, adenosine receptor)		NCT04410328 (phase 3) NCT04424901 (phase 2) NCT04391179 (phase 2)	(Ali et al., 2019; Liu et al., 2020)
**Pentoxifylline**	**PDEs**	FDA-approved	Blood flow (PDEs, adenosine receptor)		NCT04433988 (phase 1 and 2)	(Hendry et al., 2020)
**Roflumilast**	**PDE4**	FDA-approved	COPD (PDE4)			(Phillips, 2020)
**Apremilast**	**PDE4**	FDA-approved	Psoriasis		NCT04488081 (phase 2)	(Queiro Silva et al., 2020)
**CHF6001**	**PDE4**	Clinical trial		NCT02986321 (COPD, phase 2), NCT01689571 (asthma, phase 2)		(Singh et al., 2019; Phillips, 2020)
**Crisaborole**	**PDE4**	FDA-approved	Atopic dermatitis			(Hashim et al., 2020)
**Ensifentrine (RPL554)**	**PDE4**	Clinical trial		NCT04027439 (COPD, phase 2)	NCT04527471 (phase 2)	(Cazzola et al., 2019)
**Disulfiram**	**GSDMD** (NETs)	FDA-approved	Chronic alcoholism (aldehyde dehydrogenase)		NCT04485130 (phase 2)	(Lobo-Galo et al., 2020)
**Dornase alfa (Pulmozyme, rhDNase I)**	**DNAse** (NETs)	FDA-approved	CF (DNA)		NCT04402970 (phase 3) NCT04359654 (phase 2) NCT04355364 (phase 3) EudraCT 2020-001492-33 (phase 3) and 5 more	(Weber et al., 2020)
**BMS-986253 (Humax IL-8)**	**IL-8** (mAb)	Clinical trial		NCT03400332 (cancer, phase 1 and 2)	NCT04347226 (phase 2)	(Bilusic et al., 2019)
**AZD5069**	**CXCR2** (receptor for IL-8)	Clinical trial		NCT01255592 (bronchiectasis, phase 2)		(Nicholls et al., 2015; Cullberg et al., 2018)
**Danirixin (GSK1325756)**	**CXCR2** (receptor for IL-8)	Clinical trial		NCT03034967 (COPD, phase 2)		(Madan et al., 2019; Barth et al., 2020; Lazaar et al., 2020)
**Navarixin (SCH527123)**	**CXCR2** (receptor for IL-8)	Clinical trial		NCT00688467 (allergen-induced asthma, phase 2)		(Holz et al., 2010; Todd et al., 2016)
**Ixekizumab**	**IL-17A** (mAb)	FDA-approved	Psoriasis (IL-17A)			(Bulat et al., 2020)
**Secukinumab**	**IL-17A** (mAb)	FDA-approved	Autoimmune diseases, RA, and psoriasis (IL-17A)		NCT04403243 (phase 2) EudraCT 2020-001246-18 (phase 4)	(Bulat et al., 2020)
**Brodalumab**	**IL-17A receptor** (mAb)	FDA-approved	Autoimmune diseases, RA, and psoriasis (IL-17A receptor)			(Bulat et al., 2020)
**Anakinra**	**IL-1** receptor (mAb)	FDA-approved	Autoimmune diseases (IL-1)		NCT04330638 (phase 3) NCT04364009 (phase 3) EudraCT 2020-001963-10 (phase 3) and 14 more	(Van De Veerdonk and Netea, 2020)
**Canakinumab**	**IL-1β** (mAb)	FDA-approved	Autoimmune diseases (IL-1)		NCT04362813 (phase 3) EudraCT 2020-001370-30 (phase 3) and 4 more	(Prieto-Peña and Dasgupta, 2020)
**Rilonacept**	**IL-1β** (mAb)	FDA-approved	Autoimmune diseases (IL-1)			(Prieto-Peña and Dasgupta, 2020)
**Tocilizumab**	**IL-6 receptor** (mAb)	FDA-approved	Autoimmune diseases (IL-6 receptor)		NCT04317092 (phase 2) NCT04320615 (phase 3) EudraCT 2020-001903-17 (phase 3) and 38 more	(Guaraldi et al., 2020)
**Sarilumab (Kevzara)**	**IL-6 receptor** (mAb)	FDA-approved	RA (IL-6 receptor)		NCT04315298 (phase 2 and 3) EudraCT 2020-001531-27 (phase 2) and 10 more	(Lu et al., 2020)

^1^Updated on 1^st^ September 2020 via https://clinicaltrials.gov (US national clinical trials) and https://www.clinicaltrialsregister.eu (EU clinical trials). Additional completed or ongoing clinical trial studies in COVID-19 using the drug as main intervention are indicated. ARDS, acute respiratory distress syndrome; CF, cystic fibrosis; COPD, chronic obstructive pulmonary disease; CXCR2, chemokine receptor 2; DPP1, dipeptidyl peptidase 1; GSDMD, gasdermin D; IL, interleukin; mAb, monoclonal antibody; NE, neutrophil elastase; NET, neutrophil extracellular trap; PDE, phosphodiesterase; RA, rheumatic arthritis; ROS, reactive oxygen species.

## General Characteristics of COVID-19 Associated ARDS

**Figure 1:**
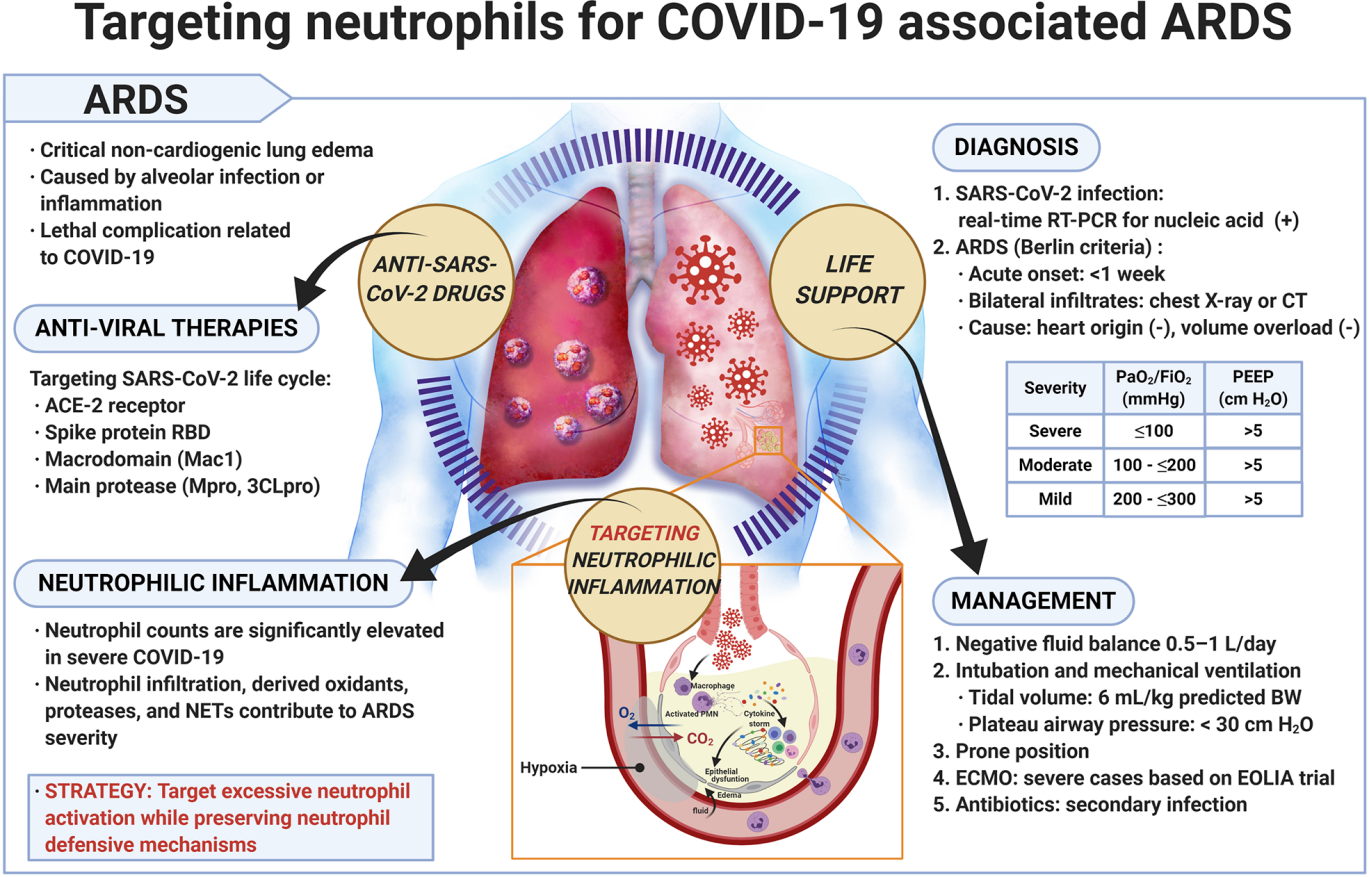
Targeting neutrophils for COVID-19 associated ARDS. Precise diagnosis of SARS-CoV-2 infection and effective management of COVID-19 are important. Application of antiviral therapy together with suppression of overly active neutrophilic inflammation appears to be a promising strategy for treating patients with COVID-19 associated ARDS. ARDS, acute respiratory distress syndrome; BW, body weight; COVID-19, coronavirus disease 2019; CT, computed tomography; ECMO, extracorporeal membrane oxygenation; EOLIA, ECMO to rescue lung injury in severe ARDS; FiO_2_, inspired oxygen fraction; ACE2, angiotensin-converting enzyme 2; NET, neutrophil extracellular trap; PaO_2_, arterial oxygen tension; PEEP, positive end-expiratory pressure; PMN, polymorphonuclear leukocyte; RBD, receptor binding domain; RT-PCR, reverse transcription polymerase chain reaction; SARS-CoV-2, severe acute respiratory syndrome coronavirus 2.

ARDS is a critical noncardiogenic pulmonary edema caused by alveolar infection or inflammation. Patients who develop ARDS suffer from a series of nonspecific manifestations, such as cough, shortness of breath, dyspnea, tachycardia, or cyanosis of the nail bed (Sweeney and Mcauley, 2016). If respiratory failure occurs, patients require endotracheal intubation and mechanical ventilation. The mortality rate is approximately 30%–40% (Stevens et al., 2018). ARDS is diagnosed using the Berlin criteria, i.e., acute onset or worsening within one week, bilateral lung infiltrates upon chest X-ray or computed tomography scan, origin exclusive of heart failure or volume overload, disease severity based on desaturation values (severe: arterial oxygen tension/inspired oxygen fraction (PaO_2_/FiO_2_) ≤ 100 mmHg, moderate: PaO_2_/FiO_2_ 100 to ≤ 200 mmHg, and mild: PaO_2_/FiO_2_ 200 to ≤ 300 mmHg), and minimum positive end-expiratory pressure (PEEP) of 5 cm H_2_O for mechanical ventilation (**Figure 1**) (Ranieri et al., 2012). Patients with pneumonia, sepsis, gastric aspiration, or chest trauma may readily develop ARDS. Respiratory viruses, such as influenza virus, Middle East respiratory syndrome-related coronavirus (MERS), SARS-CoV, rhinovirus, respiratory syncytial virus, parainfluenza virus, human metapneumovirus, and adenoviruses may cause viral pneumonia and severe ARDS (Shah and Wunderink, 2017). SARS-CoV-2 emerged in 2019 and caused the COVID-19 outbreak. Patients with COVID-19 may experience lethal pneumonia and ARDS (Badraoui et al., 2020; Zhou P. et al., 2020). Matthay et al. provided a list of recommended treatments for patients with ARDS caused by COVID-19 including adjustment of the tidal volume to 6 ml/kg predicted weight, maintenance of the plateau airway pressure at <30 cm H_2_O, neuromuscular blockade for patient-ventilator dyssynchrony, maintenance of a prone position during ventilation for severe ARDS, maintenance of a negative fluid balance of 0.5–1.0 L/day, and antibiotic administration for secondary bacterial and fungal infections (Matthay et al., 2020). Extracorporeal membrane oxygenation (ECMO) for ARDS related to COVID-19 requires careful patient selection, intensive care, and secondary infection prevention to rescue lung injury in severe cases of ARDS (Mi et al., 2018; Ramanathan et al., 2020).

## Contribution of Neutrophils to COVID-19 Associated ARDS

SARS-CoV-2 employs human angiotensin-converting enzyme 2 (hACE2) as an entry receptor for invading host (Zhou P. et al., 2020). The hACE2 receptor is abundant in the respiratory airway, blood vessels, kidney, and intestine (Li Y. C. et al., 2020). Viral RNAs serve as pathogen-associated molecular patterns (PAMPs) and are sensed by Toll-like receptors (TLRs) such as TLR3, TLR7, TLR8, and TLR9. This results in the production of interferon α and β, along with various proinflammatory cytokines (Kawai and Akira, 2010). Lung inflammation initiated by proinflammatory macrophages and neutrophils causes ARDS, a critical issue in the severe form of COVID-19 (Shi H. et al., 2020). Patients with severe COVID-19 exhibit dysregulated immune responses, such as decreased lymphocyte levels, but increased neutrophil levels (Qin et al., 2020). The neutrophil count in patients with pneumonia was found higher than in patients with only mild acute respiratory disease related to COVID-19 (Lai et al., 2020). The remarkably elevated neutrophil count was found to serve as a marker for poor prognosis in a retrospective review of 25 deaths related to SARS-CoV-2 (Li X. et al., 2020). In another retrospective analysis of 95 patients with COVID-19, an increased neutrophil count was related to disease severity and reflected an overt inflammatory response causing complications (Zhang et al., 2020). The neutrophil-to-lymphocyte ratio was significantly elevated in patients with severe COVID-19 based on a meta-analysis. Furthermore, this ratio could be used as a marker for predicting whether more severe complications such as ARDS would arise (Lagunas-Rangel, 2020). Finally, neutrophils are suggested as a target for immunopathologic complications in severe COVID-19 patients (Tomar et al., 2020). The elevated neutrophil count in COVID-19 patients and its significant correlation with disease severity indicates the importance of neutrophils in the management of COVID-19. The elevated neutrophil count is not only an abnormal laboratory finding but also a characteristic feature that should be further evaluated to develop treatments for patients infected with SARS-CoV-2.

Cellular invasion of SARS-CoV-2 reduces hACE2 expression, thereby promoting the recruitment of neutrophils (Tomar et al., 2020). Possible strategies for developing anti-SARS-CoV-2 drugs include targeting the ACE2 receptor, the spike (S) protein receptor binding domain, the macrodomain (Mac1), and the main protease (Mpro, 3CLpro) (Alhammad et al., 2020; Ton et al., 2020; Zhu et al., 2020). Neutrophils were widespread in the alveoli in COVID-19 patients (Zuo et al., 2020). In rats, excessive neutrophil migration to the lungs caused severe lung hemorrhage and increased microvascular permeability. Therefore, neutrophils participate in viral clearance while contributing to pathological symptoms in rat respiratory coronavirus infection (Haick et al., 2014). Moreover, neutrophils play a pivotal role in the cytokine storm (Tisoncik et al., 2012). In COVID-19 patients, neutrophils secrete interleukin (IL)-6 *via* a TLR8-mediated mechanism leading to a cytokine storm and subsequent lung damage (Mohamed et al., 2020). IL-1β and NETs form a feedback loop, which contribute to the pathogenesis of ARDS in COVID-19 patients (Yaqinuddin and Kashir, 2020). SARS-CoV-2 may invade nerves and aggravate respiratory failure (Li Y.C. et al., 2020). Neutrophil reactive oxygen species (ROS) and NETs participate in demyelination of the central neural system in mice with neurological diseases and coronavirus infection (Cheng et al., 2019). Therefore, treatments targeting excessive neutrophil activation may improve pathological neutrophilic inflammation during COVID-19 infection complicated by ARDS and nerve invasion.

## Targeting Neutrophils May Improve the Treatment of ARDS Caused by SARS-COV-2 Infection

Neutrophil infiltration is the defining hallmark of ARDS (Zemans and Matthay, 2017). Elevated neutrophils and neutrophil-derived microparticles are found in bronchoalveolar lavage fluid from patients with ARDS (Nakos et al., 1998; Guervilly et al., 2011). In ARDS patients, macrophages secrete CXCL8 (IL-8) to activate a circulating neutrophil recruitment cascade *via* C-X-C chemokine receptor 1 (CXCR1) receptors. CXCL8 levels are also correlated with the severity and outcome of ARDS (Miller et al., 1992; Groeneveld et al., 1995). CXC chemokines including CXCL1/2, CXCL8, CXCL5, CXCL12, and CXCL15 are responsible for neutrophil recruitment to the lungs during lung injury. However, their blockade would not completely prevent neutrophil recruitment, which indicates a rather complicated mechanism operating during immune activation (Zemans and Matthay, 2017). The SARS-CoV S protein stimulates lung epithelial cells to release IL-8 *via* activation of MAPK and AP-1, the IL-8 promoter (Chang et al., 2004). Epithelial membrane protein 2 (Emp2) of alveolar epithelial type 1 cells is important for the regulation of neutrophil migration in ARDS. Emp2 knock-out mice displayed decreased neutrophil influx to the lungs and an improved survival rate of bacterial pneumonia (Lin et al., 2020). Therefore, anti-EMP2 diabodies may be helpful in treating ARDS by mitigating neutrophil infiltration. Moreover, CCL2 (monocyte chemoattractant protein-1, MCP-1) and CCL7 are increased in the lungs of patients with ARDS (Bhatia et al., 2012; Mercer et al., 2014). Extravasated neutrophils exhibit elevated CCL2 and CCL7 binding to CCR2 receptors. Proteinase-activated receptors (PARs) are present on epithelial cells, monocytes, macrophages, and vascular endothelial cells, and their activation leads to the release of proinflammatory mediators including the cytokines TNF, IL-1β, IL-2, and IL-6, and the chemokines CXCL8 (IL-8) and CCL2, all of which are associated with ARDS pathogenesis. Modulating the CC chemokine response *via* antagonism of PAR1 signaling or by blocking these chemokines directly, may represent a treatment model for excessive neutrophilia and tissue damage associated with ARDS (Mercer et al., 2014). The level of neutrophil-derived calprotectin, along with other acute inflammatory markers, is correlated with pulmonary severity caused by SARS-CoV-2, which indicates that neutrophils are drivers of the thrombo-inflammatory storm, not just bystanders (Shi Y. et al., 2020). Mitochondrial formyl peptides are elevated in ARDS patients (Dorward et al., 2017) and formyl peptides are known to drive neutrophils in ARDS. Formyl peptide receptors (FPRs) play an important role in the activation of neutrophils (Yang and Hwang, 2016; Chen et al., 2017), and FPR-1 expression is elevated in lung injury and fibrosis (Leslie et al., 2020). Several FPR1 antagonists were discovered previously in our lab including the clinical drug propofol (Yang et al., 2013; Liu et al., 2017; Yang et al., 2017; Chen et al., 2018) that may have potential in the development of the treatment for COVID-19 associated ARDS.

NETs are composed of sticky chromatin decorated with various granular components (Zawrotniak and Rapala-Kozik, 2013). Interestingly, sputum viscosity is correlated with the level of NETs (Papayannopoulos et al., 2011; Manzenreiter et al., 2012). Mucokinetic drugs that preserve viscoelasticity, not mucolytics, were recommended for the management of cystic fibrosis (Henke and Ratjen, 2007). This could also be applied to ARDS in COVID-19 patients. NET level was found related to the polarization of proinflammatory M1 macrophages in ARDS patients; furthermore, NET inhibitors repressed NET formation and reduced M1 macrophage markers in a mouse model of acute lung injury (Song et al., 2019). Phagocytosis of NETs by macrophages is impaired in ARDS (Grégoire et al., 2018). Activation of the AMP-activated protein kinase (AMPK) pathway stimulates macrophage efferocytosis (Bae et al., 2011). Therefore, drugs interacting with AMPK, such as metformin, may reduce ARDS severity (Grégoire et al., 2018). Moreover, NET levels in the plasma are known to be correlated with ARDS mortality (Lefrançais et al., 2018). NETs consist of neutrophil-extruded DNA coated with histones, neutrophil elastase (NE), and myeloperoxidase (MPO). Peptidylarginine deiminase 4 (PAD4), NE, and gasdermin D along with free DNA, all participate in the NET formation (Nathan, 2020). NETs are prevalent in blood, trachea, and lung specimens of COVID-19 patients (Veras et al., 2020). Moreover, SARS-CoV-2 stimulates neutrophils from healthy donors into forming NETs, which can cause apoptosis in respiratory epithelial cells *in vitro* (Oubihi and Wang, 2020). High levels of plasma MPO-DNA complex and aberrant NET formation are correlated with severe ARDS in COVID-19 (Middleton et al., 2020; Zuo et al., 2020). Also, elevated levels of cell-free DNA were observed in COVID-19 patients (n = 50), as well as highly specific markers of NETs, such as MPO-DNA and citrullinated histone H3 (Cit-H3), along with other typical markers (C-reactive protein, D-dimer, neutrophil count, etc.) (Zuo et al., 2020). NETs contributed to microthrombi through platelet-neutrophil interactions in COVID-19 associated ARDS, and neonatal NET-Inhibitory Factor (nNIF) could block NET formation induced by COVID-19 plasma. This represents a potential therapeutic intervention for COVID-19 (Middleton et al., 2020). PAD4 is a predominant driver of histone citrullination in NETs (Wong and Wagner, 2018). Currently, several PAD4 inhibitors were demonstrated to inhibit NET formation *in vitro*. Among them, Cl-amidine (Kusunoki et al., 2016), GSK484 (Lewis et al., 2015), and BMS-P5 (Li M. et al., 2020) may have future development potential. Therefore, inhibition of neutrophil activation and NET formation may be beneficial in COVID-19-associated ARDS.

## Drugs Targeting Neutrophils for COVID-19 ASSOCIATED ARDS

**Figure 2:**
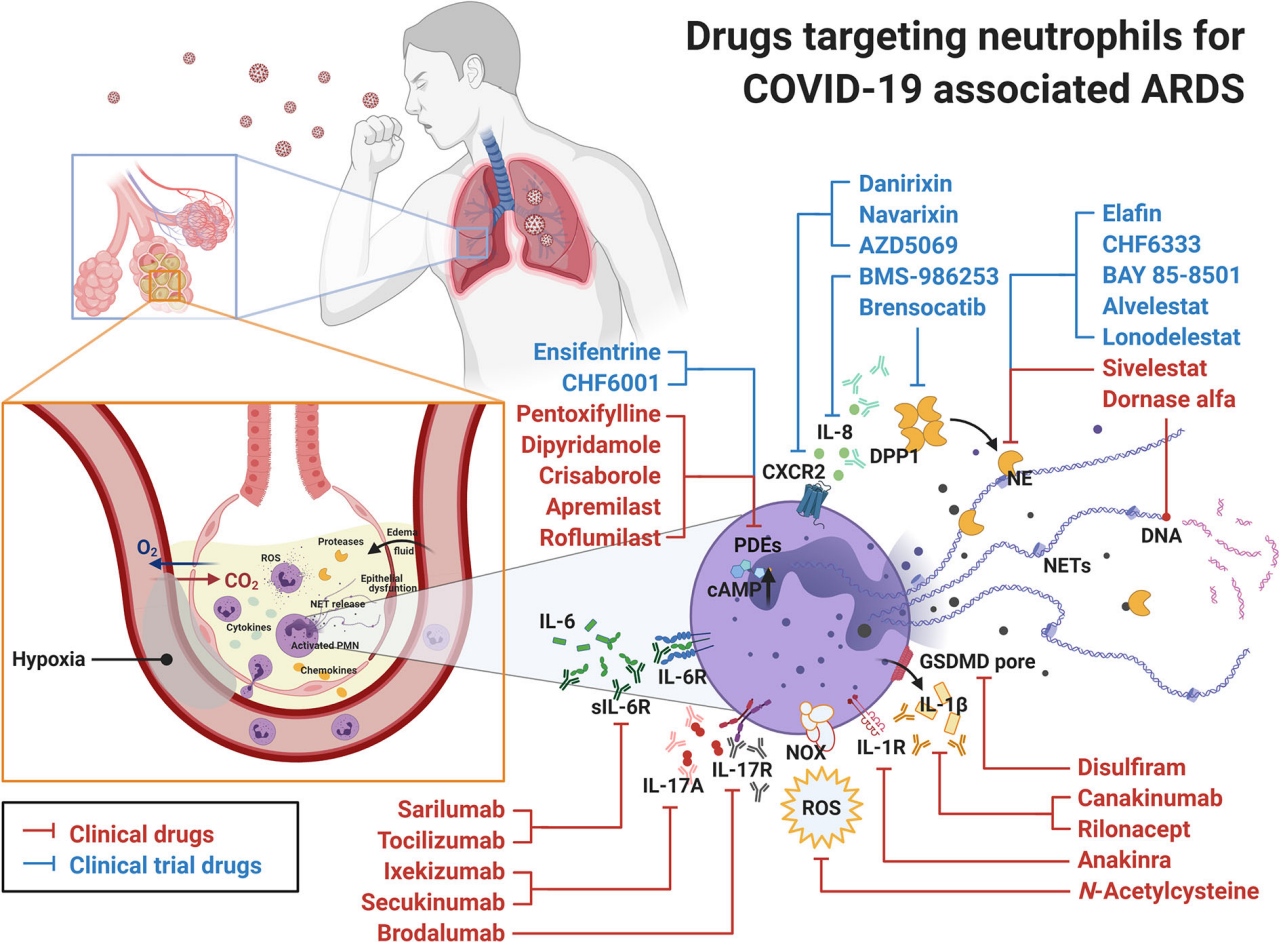
Drugs targeting neutrophils for COVID-19 associated ARDS. Clinical drugs (in red) and clinical trial drugs (in blue) are displayed with their targets in neutrophils. In COVID-19 infection, the release of SARS-CoV-2 RNA in lungs serves as PAMP and induces complicated immune reactions leading to ARDS and respiratory failure. Under these circumstances, neutrophils recruited to the site of infection participate in the elimination of SARS-CoV-2, but they also contribute to the pathogenesis of ARDS. Therefore, the commercial drugs targeting neutrophils might be considered as novel candidates to treat COVID-19 associated ARDS. ARDS, acute respiratory distress syndrome; CXCR, C-X-C chemokine receptor; DPP1, dipeptidyl peptidase 1; GSDMD, gasdermin D; IL, interleukin; NE, neutrophil elastase; NET, neutrophil extracellular trap; NOX, NADPH oxidase; PDE, phosphodiesterase; PMN, polymorphonuclear leukocyte; ROS, reactive oxygen species.

Currently, there are several clinical drugs indicated for use in respiratory diseases that affect neutrophil function, but other drugs should also be considered for the treatment of ARDS in COVID-19. A summary of commercially available approved drugs or those in clinical trials, allocated to different groups based on their specific target in neutrophils, is provided (**Figure 2**, **Table 1**).

### Neutrophil Elastase Inhibitors

Neutrophil elastase (NE) contributes to the invasion of SARS-CoV-2 into host cells and can also damage lung tissue directly, thus participating in the pathogenesis of COVID-19 associated ARDS (Thierry, 2020). Moreover, NE is an important component and plays an important role in NETosis. For instance, the administration of elastase inhibitors such as sivelestat (Kim et al., 2014) and BAY 85-8501 (Von Nussbaum et al., 2015) ameliorated acute lung injury in mice. Currently, sivelestat is the only approved NE inhibitor for the treatment of ARDS in Korea and Japan (Aikawa and Kawasaki, 2014). There are several other NE inhibitors in different stages of clinical trials including alvelestat (AZD9668, COPD, phase 2, US national clinical trial number NCT03636347) (Stockley et al., 2013), BAY 85-8501 (noncystic fibrosis bronchiectasis, phase 2, NCT01818544), (Watz et al., 2019), lonodelestat (POL6014, cystic fibrosis, phase 1, NCT03748199) (Barth et al., 2020), CHF6333 (noncystic fibrosis bronchiectasis, phase 1, NCT03056326, NCT04010799) (Gramegna et al., 2017), and elafin (ARDS, phase 1, NCT02944279) (Barnes et al., 2020). In a meta-analysis study, sivelestat failed to improve the survival of patients with ARDS (Tagami et al., 2014). However, a retrospective cohort study with 66 ARDS patients demonstrated that sivelestat treatment yields positive outcomes (Maki et al., 2020). In particular, aerosol- or nebulizer-dosed NE inhibitors significantly improved their efficacy and lowered adverse effects (Barth et al., 2020). Thus, prompt administration of NE inhibitors may be helpful in severe COVID-19 patients with ARDS (Mohamed et al., 2020).

### Respiratory Burst Inhibitor

*N*-Acetylcysteine is a potential therapeutic in the treatment of COVID-19 (Andreou et al., 2020). It is a mucolytic drug with antioxidant activity that is used in respiratory diseases (Mokhtari et al., 2017) and skin diseases (Adil et al., 2018), as well as an antidote in acetaminophen overdose (Mokhtari et al., 2017). Moreover, *N*-acetylcysteine inhibited the respiratory burst in activated neutrophils *in vitro* (Allegra et al., 2002) and in patients in the intensive care unit (Heller et al., 2001). Furthermore, *N*-acetylcysteine alleviated acute lung injury *in vivo* under various conditions (Alkan et al., 2006; Chuang et al., 2007; Liu et al., 2008; Yubero et al., 2012; Guo et al., 2019), and inhibited lung fibrosis *in vivo* (Kulshrestha et al., 2020) with limited patient outcomes, according to a meta-analysis study (Sun et al., 2016). In another meta-analysis study, *N*-acetylcysteine treatment of ARDS patients shortened their stay in the intensive care unit (Zhang et al., 2017). Finally, it has also been suggested that *N*-acetylcysteine should be used in combination with other drugs to manage ARDS (Guo et al., 2019; Andreou et al., 2020; Horowitz and Freeman, 2020). Currently, several clinical trials with *N*-acetylcysteine to treat COVID-19 (NCT04419025, NCT04455243, NCT04374461, etc.) are ongoing. Based on the above, we believe that *N*-acetylcysteine may be helpful in treating ARDS caused by SARS-CoV-2 *via* its antioxidant and anti-respiratory burst activity.

### Dipeptidyl Peptidase 1 Inhibitor

Dipeptidyl peptidase 1 (DPP1), also known as cathepsin C, is a cysteine dipeptidyl aminopeptidase that activates serine proteases such as NE during maturation of neutrophils. Excessive serine protease activity causes various inflammatory lung diseases such as ARDS and contributes to COPD and asthma. Brensocatib (also called INS1007 or AZD7986), a DPP1 inhibitor, was found to reduce NE activity in healthy humans (Palmér et al., 2018) and is now in clinical trials for bronchiectasis (NCT03218917) and COVID-19 (EU clinical trial number EudraCT 2020-001643-13). The administration of DPP1 inhibitors may prevent ARDS progression caused by SARS-CoV-2 (Korkmaz et al., 2020). Thus, DPP1 inhibitors are of interest in treating COVID-19 associated ARDS.

### PDE4 Inhibitors

Phosphodiesterases (PDEs) belong to the class of enzymes that metabolize the intracellular second messenger cyclic adenosine monophosphate (cAMP) and cyclic guanosine monophosphate (cGMP). In particular, cAMP-specific PDE4 type is widely present in immune cells including neutrophils and contributes to neutrophil-mediated lung inflammation (Baillie et al., 2019). There are currently three FDA-approved PDE4 inhibitors: roflumilast for COPD (Phillips, 2020), apremilast for psoriasis (Queiro Silva et al., 2020), and crisaborole for atopic dermatitis (Hashim et al., 2020). Other drugs such as CHF6001 (NCT02986321 for COPD and NCT01689571 for asthma) (Singh et al., 2019), and ensifentrine (RPL554, NCT04027439 for COPD) (Cazzola et al., 2019) are awaiting phase 3 clinical trials. However, many of the experimental drugs were discontinued in clinical trials due to side effects (Phillips, 2020). Several PDE inhibitors have been proposed to be suitable drugs for COVID-19 treatment (Giorgi et al., 2020). Thus, we suggest their clinical consideration in COVID-19 associated ARDS. In light of the above, clinical trials targeting COVID-19 using apremilast (NCT04488081) or ensifentrine (NCT04527471) have been initiated.

Dipyridamole is an FDA-approved nonspecific PDE inhibitor used for thrombosis and was discovered to inhibit NET formation (Ali et al., 2019). Dipyridamole acts by increasing intracellular cAMP levels and blocking adenosine reuptake in cells, thereby leading to its antiplatelet and vasodilatory effects (Tan et al., 2019). In a trial including 31 COVID-19 patients, dipyridamole showed improvement in severe cases with significantly reduced D-dimer levels (Liu et al., 2020). Currently, several COVID-related trials are ongoing for dipyridamole (NCT04410328, NCT04424901, NCT04391179). Another FDA-approved nonspecific PDE inhibitor, pentoxifylline, is a derivative of caffeine. Pentoxifylline stimulates blood flow, inhibits platelets, and has immunomodulatory and anti-inflammatory properties. Pentoxifylline also inhibits neutrophil adhesion (Bone, 1992). It is currently in ongoing clinical trials for COVID-19 (NCT04433988). Along with specific PDE4 inhibitors, dipyridamole and pentoxifylline may represent suitable candidates for further anti-COVID-19 development (Hendry et al., 2020).

### Gasdermin D Inhibitor

Disulfiram, an FDA-approved gasdermin D inhibitor, blocks SARS-CoV-2 replication *in silico* (Lobo-Galo et al., 2020). Moreover, disulfiram was shown to abrogate gasdermin D pore formation by covalent bonding to Cys191/Cys192 (Hu et al., 2020). Gasdermin D is important in the formation of NETs (Sollberger et al., 2018). Therefore, disulfiram has the potential to reduce NET-related pathogenesis in ARDS caused by SARS-CoV-2. Currently, a clinical trial is ongoing on the potential use of disulfiram in COVID-19 (NCT04485130).

### DNase Inhibitors

Application of DNase I to mice with severe bacterial pneumonia and acute lung injury reduced NET formation and improved their survival rate (Lefrançais et al., 2018). Administration of dornase alfa, an FDA-approved recombinant human DNAse I, using a nebulizer in severe COVID-19 associated ARDS patients may help lyse the sputum and improve disease progression (Barnes et al., 2020; Weber et al., 2020). Currently, there are several COVID-19 clinical trials using dornase alfa (NCT04402970, NCT04359654, NCT04355364, EudraCT 2020-001492-33, etc.) (Desilles et al., 2020). Another promising DNase inhibitor, AIR DNAse™, is currently undergoing phase 2 clinical trials in cystic fibrosis patients (NCT02605590, NCT02722122).

### Chemokine-Related Drugs (IL-8, IL-17, IL-1β and IL-6)

IL-8 secreted by macrophages and lung epithelial cells is a neutrophil chemoattractant. Moreover, IL-8 contributes to neutrophil activation and NET formation after binding to CXCR2 on neutrophils, thereby causing hyperinflammation. Interestingly, anti-IL-8 monoclonal antibody BMS-986253 (Humax IL-8), developed as an anti-tumor treatment (Bilusic et al., 2019), is currently in clinical trial for COVID-19 (NCT04347226). AZD5069 (Nicholls et al., 2015; Cullberg et al., 2018), danirixin (Madan et al., 2019; Lazaar et al., 2020), and navarixin (SCH527123) (Holz et al., 2010; Todd et al., 2016) are available CXCR2 inhibitors that ameliorate neutrophil activation in pulmonary diseases including bronchiectasis, virus-associated lung infection, COPD and asthma. Therefore, they may represent valuable drugs for the treatment of ARDS in COVID-19 patients (Narasaraju et al., 2020). However, the development of the CXCR2 antagonist QBM076 was terminated for safety reasons (NCT01972776).

IL-17A is a proinflammatory cytokine involved in inflammation and immune responses; thus, blocking its effect is beneficial in treating neutrophilic inflammatory diseases. Ixekizumab (NCT03099538) and secukinumab (NCT03099980) are monoclonal antibodies (mAb) against IL-17A that prevent its interaction with the IL-17A receptor. In particular, IL-17A antagonists have been used for the treatment of rheumatoid arthritis and psoriasis (Chiang et al., 2019). Brodalumab (NCT04249713) binds with a high affinity to interleukin IL-17 receptor A, thereby inhibiting IL-17 proinflammatory cytokines (Bulat et al., 2020). Secukinumab is currently tested in coronavirus trials (NCT04403243, EudraCT 2020-001246-18).

Anakinra is a IL-1 receptor inhibitor, while canakinumab and rilonacept are IL-1β inhibitors (Prieto-Peña and Dasgupta, 2020). Anakinra was reported in the treatment of COVID-19 (Van De Veerdonk and Netea, 2020). Subcutaneous administration of anakinra reduced mortality and lowered the need for advanced respiratory support in severe COVID-19 patients (Huet et al., 2020); however, several limitations were observed (Khan et al., 2020). There are currently several clinical trials in relation to COVID-19 on anakinra (NCT04330638, NCT04364009, etc.) and canakinumab (NCT04362813, etc.).

Tocilizumab, an IL-6 receptor inhibitor, reduced the level of serum C-reactive protein and ameliorated pulmonary computed tomography appearances in patients with COVID-19 (Guaraldi et al., 2020; Soy et al., 2020; Xu et al., 2020). A plethora of clinical trials using tocilizumab is currently ongoing in relation to COVID-19 (Actemra, Roche, NCT04317092, NCT04320615, etc.). Similarly, several clinical trials for another IL-6 receptor inhibitor, sarilumab (Kevzara, Regeneron, NCT04315298, etc.), have been initiated (Lu et al., 2020).

### Other Drugs

Corticosteroids are recommended by the US National Institutes of Health (NIH) for use in COVID-19 patients who are being mechanically ventilated or require oxygen supplementation (https://www.covid19treatmentguidelines.nih.gov). However, there are several concerns, and corticoids were previously not recommended in patients with ARDS related to viral pneumonia (Ni et al., 2019). Corticosteroids also reduce the level of neutrophil-derived secreted IL-1 receptor antagonist (sIL-1Ra), which leads to increased IL-1β expression (Langereis et al., 2011). Hence, corticosteroids have proinflammatory effects in neutrophils. Corticosteroids are generally thought to weakly suppress neutrophilic inflammation, although several research groups reported them to inhibit the neutrophil respiratory burst and interfere with neutrophil recruitment (Strausbaugh and Rosen, 2001; Ronchetti et al., 2018; Ricci et al., 2019). For instance, corticosteroids do not improve neutrophilic inflammation in corticosteroid-resistant asthma or COPD (Yang et al., 2012; Ronchetti et al., 2018).

Dapsone, colchicine, and olanzapine were suggested as potential adjuvant therapies in ARDS caused by SARS-CoV-2 (Altschuler and Kast, 2020). Dapsone has anti-neutrophilic activity through its inhibition of IL-8 mediated chemotaxis and thus may be therapeutic in COVID-19 associated ARDS (Farouk and Salman, 2020; Kast, 2020). Colchicine, a neutrophil microtubule polymerization inhibitor, reduces IL-1 production and is undergoing several clinical trials for COVID-19 (NCT04322682 and 18 more) (Soy et al., 2020). Olanzapine is an antipsychotic drug targeting the histamine 1 receptor, thereby reducing IL-6 generation (Altschuler and Kast, 2020).

Hydroxychloroquine and azithromycin were reported as effective anti-SARS-CoV-2 agents (Ohe et al., 2020; Zhou D. et al., 2020). Fujita et al. demonstrated that hydroxychloroquine significantly inhibited IL-1β production in activated human neutrophils *in vitro* (Fujita et al., 2019). Hydroxychloroquine inhibited NET formation and ameliorated the hypercoagulation state in mice with orthotopic pancreatic adenocarcinoma. A chemotherapy regimen with hydroxychloroquine prior to surgery improved the rate of perioperative venous thromboembolism in a clinical trial (Boone et al., 2018). With respect to the effect of azithromycin on neutrophils, Bystrzycka et al. revealed that azithromycin decreased NET release in activated neutrophils *in vitro* (Bystrzycka et al., 2017). Anderson et al. demonstrated that azithromycin inhibited neutrophil migration in dextran sulfate sodium-induced mice (Anderson et al., 2019). However, Cavalcanti et al. showed that hydroxychloroquine and azithromycin did not alter disease progression in patients with mild-to-moderate COVID-19 (Cavalcanti et al., 2020). The World Health Organization does not recommend the use of hydroxychloroquine and azithromycin due to the controversial outcomes of several trials.

## Conclusion

ARDS is the most lethal complication of COVID-19. Neutrophils, although involved in the elimination of SARS-CoV-2, also participate in the pathogenesis of COVID-19 associated ARDS. Suppression of aberrant neutrophil activation may provide an effective strategy for treating COVID-19 associated ARDS. Several clinical drugs that target neutrophils can be chosen for further therapeutic use in ARDS associated with SARS-CoV-2 infection.

## Author Contributions

C-CC, MK, and W-JC wrote the manuscript. T-LH conceived the study and edited the manuscript. All authors contributed to the article and approved the submitted version.

## Funding

This work was supported by grants from the Ministry of Science and Technology (MOST 108-2320-B-255-003-MY3, MOST 109-2327-B-255-001, and MOST 109-2327-B-182-002) and Chang Gung Memorial Hospital (BMRP450, and CORPG5K0031). The funding sources had no role in the study design; in the collection, analysis and interpretation of data; in the writing of the report; or in the decision to submit the article for publication.

## Conflict of Interest

The authors declare that the research was conducted in the absence of any commercial or financial relationships that could be construed as a potential conflict of interest.
